# Harnessing Big Data, Smart and Digital Technologies and Artificial Intelligence for Preventing, Early Intercepting, Managing, and Treating Psoriatic Arthritis: Insights From a Systematic Review of the Literature

**DOI:** 10.3389/fimmu.2022.847312

**Published:** 2022-03-10

**Authors:** Nicola Luigi Bragazzi, Charlie Bridgewood, Abdulla Watad, Giovanni Damiani, Jude Dzevela Kong, Dennis McGonagle

**Affiliations:** ^1^ Laboratory for Industrial and Applied Mathematics (LIAM), Department of Mathematics, York University, Toronto, ON, Canada; ^2^ Department of Health Sciences (DISSAL), Postgraduate School of Public Health, University of Genoa, Genoa, Italy; ^3^ Leeds Institute of Rheumatic and Musculoskeletal Medicine, University of Leeds, Leeds, United Kingdom; ^4^ Department of Medicine B, Rheumatology Unit and Zabludowicz Center for Autoimmune Diseases, Sheba Medical Center, Ramat-Gan, Israel; ^5^ Sackler Faculty of Medicine, Tel-Aviv University, Tel-Aviv, Israel; ^6^ Clinical Dermatology, Istituto di Ricovero e Cura a Carattere Scientifico (IRCCS) Galeazzi Orthopaedic Institute, Milan, Italy; ^7^ National Institute for Health Research Leeds Biomedical Research Centre, Leeds Teaching Hospitals, Leeds, United Kingdom

**Keywords:** psoriatic arthritis, big data, artificial intelligence, digital technologies, early interception, prevention

## Abstract

**Background:**

Rheumatological and dermatological disorders contribute to a significant portion of the global burden of disease. Big Data are increasingly having a more and more relevant role, being highly ubiquitous and pervasive in contemporary society and paving the way for new, unprecedented perspectives in biomedicine, including dermatology and rheumatology. Rheumatology and dermatology can potentially benefit from Big Data.

**Methods:**

A systematic review of the literature was conducted according to the “Preferred Reporting Items for Systematic Reviews and Meta-Analyses” (PRISMA) guidelines, mining “Uno per tutti”, a highly integrated and automated tool/meta-database developed at the University of Genoa, Genoa, Italy, and consisting of 20 major scholarly electronic databases, including PubMed/MEDLINE. Big Data- or artificial intelligence-based studies were judged based on the modified Qiao’s critical appraisal tool for critical methodological quality assessment of Big Data/machine learning-based studies. Other studies designed as cross-sectional, longitudinal, or randomized investigations, reviews/overviews or expert opinions/commentaries were evaluated by means of the relevant “Joanna Briggs Institute” (JBI)’s critical appraisal tool for the critical methodological quality assessment.

**Results:**

Fourteen papers were included in the present systematic review of the literature. Most of the studies included concerned molecular applications of Big Data, especially in the fields of genomics and post-genomics. Other studies concerned epidemiological applications, with a practical dearth of studies assessing smart and digital applications for psoriatic arthritis patients.

**Conclusions:**

Big Data can be a real paradigm shift that revolutionizes rheumatological and dermatological practice and clinical research, helping to early intercept psoriatic arthritis patients. However, there are some methodological issues that should be properly addressed (like recording and association biases) and some ethical issues that should be considered (such as privacy). Therefore, further research in the field is warranted.

**Systematic Review Registration:**

Registration code 10.17605/OSF.IO/4KCU2.

## Introduction

The global burden of disease (GBD) is the quantitative assessment of the health loss due to a given disorder, risk factor, or injury, over a span of time, worldwide. It is comprehensively, geo-spatially and temporally modeled and computed as the epidemiological, clinical, and societal burden imposed by a given disease, taking into account its economic-financial and humanistic effects, if inadequately managed and treated. Such a quantitative and broad approach enables practitioners and researchers as well as all relevant stakeholders, including public and global health decision- and policymakers, to consistently compare the burden of different diseases, risk factors, or injuries, over time and across countries.

Furthermore, these data can guide health policies, guiding them in a pure data-driven and evidence-based way, enabling them to prioritize and allocate resources, especially in developing countries and in other resource-limited settings (1). This framework allows to track the impact of a given health policy or medical intervention (pharmaceutical or non-pharmaceutical/surgical) and to verify if satisfactory progress has been attained towards the achievement of the Sustainable Development Goals (SDGs) set up by the United Nations (UN) General Assembly (2). In particular, SDG 3.4.1 has proposed the ambitious goal of achieving a 30% reduction in premature mortality due to non-communicable diseases by 2030 (2).

In order to monitor the achievement of such a target, the GBD initiative as well as other similar taskforces and groups, like the Global Health Estimates (GHE) initiative led by the World Health Organization (WHO), have designed and validated an array of reliable health-related metrics. These indicators include the number of years of life lost (YLLs), the number of years lived with disability (YLDs), and the number of disability-adjusted life years (DALYs), which enable scholars to compute life lost due to death (because of casualty or premature death) or disability, respectively, which do not allow to live life at maximum (100%) health (1).

GBD- and GHE-related measures are of crucial importance in providing stakeholders involved in the field of global and public health with data, especially in those settings and territories where there is a lack of data, or data are not updated and/or of good quality, in that data collection and analysis would be too much time- and resource-consuming (1).

Psoriasis is a chronic inflammatory skin disease, the complex, multi-factorial etiopathogenesis of which has yet to be elucidated in detail, and which significantly contributes to the GBD (3).

According to a recent study conducted according to the GBD 2019 methodology (4), there were more than 4.6 million incident cases of psoriasis worldwide in 2019, with an age-standardized incidence rate of 57.8 per 100,000 people. Compared to 1990, this corresponded to a reduction of 20.0%. By sex, the age-standardized incidence rate was comparable between men and women. Compared to 1990, this corresponded to a reduction by 19.5% and by 20.4%, respectively. The age-standardized incidence rate per 100,000 persons widely varied across geographic settings, with high-income countries and territories reporting the highest rate of psoriasis, followed by high-middle income countries. Similar patterns could be found for the other GBD-related metrics, including prevalence and YLDs.

Up to approximately 30% of psoriatic patients develop psoriatic arthritis, which can result in irreversible joint damage. According to a recently published systematic review of the literature and meta-analysis (5), the degree of psoriasis severity and nail pitting were found to be predictors of early-onset psoriatic arthritis insurgence and development, as well as higher body mass index and a family history of psoriatic arthritis. Psoriatic patients with arthralgia (overall relative risk or RR 2.15 [95% confidence interval or CI ranging from 1.16 to 3.99]) and/or with imaging-musculoskeletal inflammation (pooled RR 3.72 [95%CI 2.12 to 6.51]) were deemed at higher risk for developing psoriatic arthritis.

Early detection and interception of psoriatic arthritis in psoriatic patients would be of paramount importance for ensuring timely, effective treatment and management but is rather challenging for dermatologists to implement in their daily clinical practice (5).

The way healthcare provisions are delivered has profoundly changed in the last years, with the emergence of novel and innovative models and paths of managing and treating disorders. A new, holistic, and comprehensive biomedical framework known as “P4 medicine” (where the 4 Ps stay for preventative, predictive, personalized, and participatory) has been introduced by Doctor Leroy Hood, a pioneering and inspiring figure in the arena of systems biology and systems medicine, indicating the paradigm change from a “one-size-fits-all” approach to one in which the individual needs of each patient are taken into account (6–9).

Moreover, thanks to its latest scientific achievements and technological improvements, medicine, including dermatology and rheumatology (9), is entering a new, unprecedented era, characterized by the production and release of an incredible wealth of data, known as Big Data. They are characterized by several key dimensions and features, which include velocity (Big Data or “Fast Data” can be generated, mined, retrieved, processed and analyzed in real-time and can be deployed for nowcasting/forecasting predictions), volume (related to the massive amount of data, the magnitude of which poses serious challenges to the classical ways of storing and processing data, requiring cutting-edge and powerful analytical capacities and infrastructures), variety (referring to the incredible diversity of data sources, ranging from administrative to patient-reported, healthcare-generated data, etc.), veracity (trustworthiness, credibility, reliability, and accuracy of data), and value (related to the transformation from raw “useless” data to smart, applicable, and actionable data) (10).

Different tools, channels, and sources can produce Big Data: from large-scale, nationwide surveys, databases, repositories, and registries (the so-called epidemiological/clinical Big Data) to wet-lab, next-generation sequencing and high-throughput technologies (molecular Big Data) and computational approaches (infodemiological or digital Big Data) (10).

Big Data, smart and digital technologies, and Artificial intelligence are dramatically transforming research and clinical practices into disruptive ones, being informed, and guided by purely data-driven approaches (10–12).

As outlined in a commentary by Scarpa (13), psoriatic arthritis presents a certain degree of clinical complexity, overlapping with other rheumatological diseases, on the one hand, but, on the other hand, having its own features (etiopathological, biological, clinical, epidemiological, diagnostic, prognostic, and therapeutic). To reflect such complexity, the expressions “psoriatic disease” (13) and “psoriatic syndrome” (14) have been introduced in the scholarly literature. A contemporary, rather sophisticated conceptual framework distinguishes between phenotypes (“cluster of visible features and properties”), genotypes, endotypes (“various disease expressions in patient groups depending on different disease mechanisms”), regiotypes (“regional differences among endotypes due to environmental exposures”), inflatypes (“different inflammatory mechanisms among endotypes”), and theratypes (“targeted therapeutics depending on the specific disease endotype”) (15). All such complexity and dynamic variability require a multidisciplinary approach for effective treatment and management of psoriatic disease, establishing clinical networks and models of close dermatology-rheumatology collaborations (16).

**Figure 1:**
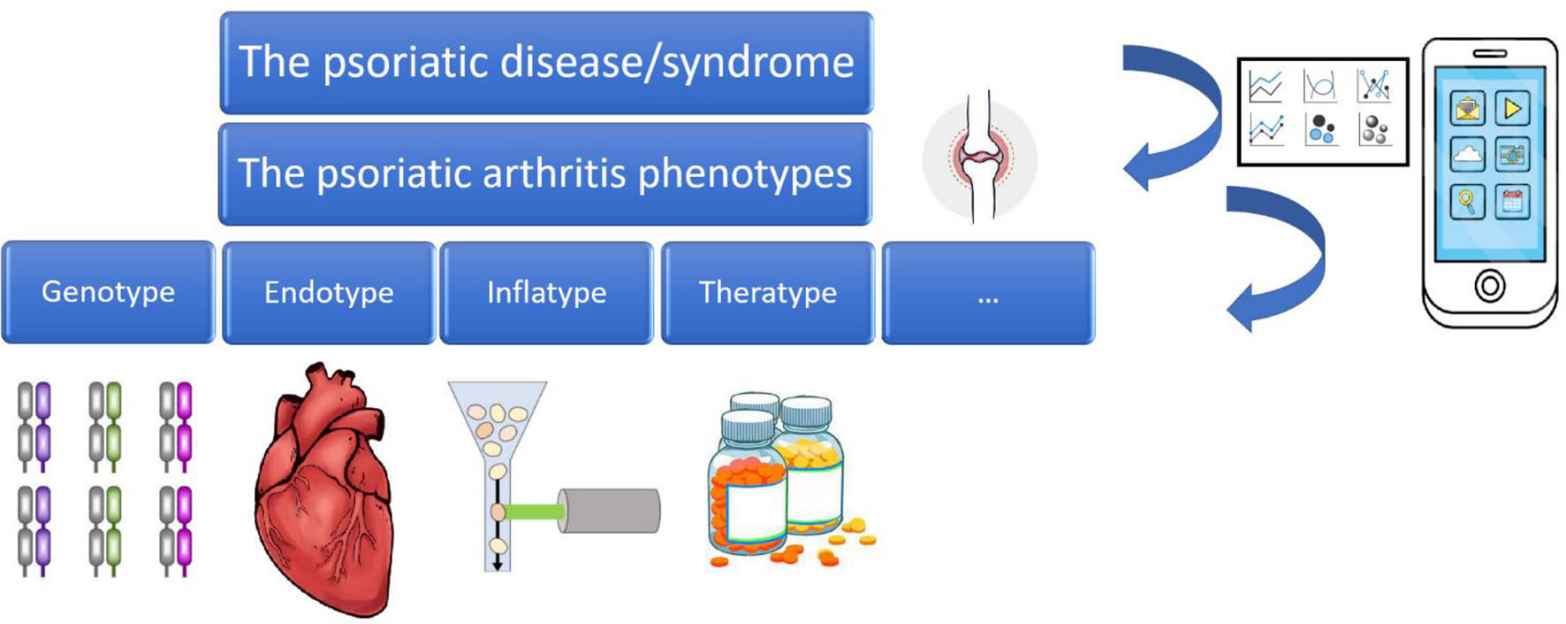
The psoriatic disease/syndrome and its various phenotypes and endotypes, leveraged by Big Data, smart and digital technologies, and Artificial Intelligence.

In the present review paper, we will show how dermatology and rheumatology can benefit from the use of the so-called “Big Data”, smart and digital technologies, and artificial intelligence, especially in the efforts of early intercepting and mitigating against the burden of psoriatic arthritis. More specifically, the present paper will provide a brief overview on the potential uses of Big Data in the field of “precision rheumatology” and “precision dermatology” (11, 12), focusing on psoriatic arthritis, broken down according to tool/source/channel, showing practical examples and applications of Big Data, as well as their major shortcomings and limitations. Big Data, smart and digital technologies, and artificial intelligence can be leveraged to dissect the clinical complexity underlying the conceptual framework of “psoriatic disease”, paving the way for personalized treatment of psoriatic arthritis (17, 18) (**Figure 1**).

## Material and Methods

### Study Protocol and Reporting of the Findings

The findings of the present systematic review are reported according to the “Preferred Reporting Items for a Systematic Review and Meta-Analysis” (PRISMA) 2020 guidelines (19). The study protocol was registered within the “Open Science Framework” (OSF, registration code 10.17605/OSF.IO/4KCU2).

### Literature Search

A string of *ad hoc* keywords was utilized, including terms such as “big data”, “artificial intelligence”, “machine learning”, “deep learning”, “random forests”, “support vector machine”, “artificial neural networks”, “natural language processing”, “k-nearest neighbors”, “Bayesian model”, “digital health”, mHealth, eHealth, uHealth, “psoriatic arthritis”, prevention, and “early interception”. This search string was designed based on a comprehensive systematic literature review conducted on the current status of the use of big data and artificial intelligence in rheumatic and musculoskeletal diseases (RMDs) to inform the “European Alliance of Associations for Rheumatology” (EULAR) recommendations (20). Moreover, extensive cross-referencing was applied in order to maximize the chance of getting all potentially eligible and relevant studies.

### Databases Mining

A highly automated and integrated discovery tool/meta-database consisting of 20 scholarly electronic databases, including PubMed/MEDLINE, named as “Uno per tutti”, developed at the University of Genoa, Genoa, Italy, was mined from inception, without time or language filters and restrictions. Wild-card option and “medical subject headings” (MeSH) terms were used when necessary. An experienced librarian and research methodologist from the University of Genoa, Genoa, Italy, were involved in the design and implementation of the search strategy.

### Inclusion and Exclusion Criteria

Inclusion and exclusion criteria were devised and formulated according to the PICOS components.

Inclusion criteria were the following: namely, i) studies written in any language, ii) original investigations applying sophisticated statistical or artificial intelligence-based techniques or relying on big data (C, comparators), and focusing on psoriatic arthritis (P, patients), or reviews on the topic under study (S, study design). Any kind of intervention (diagnostic test, pharmacological treatment, telehealthcare provision, etc.) was considered for eligibility. The feasibility and the potential effectiveness of exploiting Big Data, Artificial intelligence, and smart technologies were the major outcomes (O) of the present study. Articles that did not meet with these inclusion criteria were excluded.

Inclusion and exclusion criteria are further detailed in **Table 1**.

**Table 1:** Inclusion and exclusion criteria.

PICOS components	Inclusion criteria	Exclusion criteria
P (patients)	Psoriatic arthritis patients or psoriatic patients at higher risk for developing psoriatic arthritis	Patients with other rheumatological/dermatological conditions
I (interventions)	Any kind of intervention (diagnostic test, pharmacological treatment, eHealth/mHealth/telehealthcare provision, etc.)	None
C (comparators)	Different Big Data analytical techniques; Big Data analytical approaches versus conventional approaches	Other kinds of comparators (for example, other rheumatological/dermatological conditions)
O (outcomes)	Effectiveness of exploiting Big Data, Artificial Intelligence in early intercepting psoriatic arthritis patients, as well as in the prevention, management, and treatment of psoriatic arthritis	Other outcomes (for example, clinical, not related to the research question)
S (study design)	Any study design (original cross-sectional, longitudinal, randomized investigation, review, overview, expert opinion, commentary, etc.), with sufficient details, without time or language filter/restriction	Study with insufficient details

### Data Abstraction

Two independent researchers (N.L.B. and C.B.) abstracted relevant data from included studies. These data included: the surname of the first author of the study, study design, study country, study period, sample size, and main findings. Any potential disagreement between the two authors was solved involving a third author (D.M-G), who acted as a final referee.

### Data Synthesis

Data abstracted were collected and provided in a tabular fashion. A qualitative thematic synthesis was offered, based on the major topics addressed and covered by the retained studies.

### Methodological Quality Appraisal

Studies retained in the present systematic literature review were critically appraised in terms of methodological quality according to relevant checklists and guidelines. Big Data- and artificial intelligence/machine learning-based studies were judged based on a modified version of the recently proposed Qiao’s checklist (21) (**Supplementary Table 2**). This checklist consists of nine categories/domains: namely, i) unmet needs, ii) reproducibility, iii) robustness, iv) stability of results, v) generalizability, vi) clinical significance, and vii) suggested clinical use. The first category/domain comprises of a single item (“limits in current non-Big Data/machine learning approaches”), including low diagnostic/predictive accuracy or particularly time- and resource-consuming diagnostic procedure. The second category/domain comprises of three items: namely, i) feature engineering methods/parameters choice (feature generated before model training/identification and choice of predictors/covariates), ii) platforms and/or packages utilized, including details of databases and the mining step performed, and iii) hyperparameters/meta-data needed for study replication and confirmation. The third category/domain comprises of a single item: validated, reliable methods (such as leave-one-out, k-fold cross-validation or bootstrapping) used to overcome the issue of over-fitting. The fourth category/domain consists of a single item: computed/estimated variation during the validation step. The fifth category/domain comprises of a single item: external data validation, that is to say, validation in settings/datasets/databases different from the original setting(s)/dataset(s)/database(s) of the research framework. The last category/domain consists of two items: namely, i) explanation of the importance of each predictor/covariate, and ii) suggested potential applications and uses during routine daily clinical practice. Studies that reported at least 4 “yes” out of 9 “yes” for the modified Qiao’s checklist were considered acceptable.

Other studies designed as cross-sectional, longitudinal, or randomized investigations, reviews/overviews or expert opinions/commentaries were evaluated by means of the relevant “Joanna Briggs Institute” (JBI)’s critical appraisal tool for the critical methodological quality assessment.

Two authors (N.L.B. and C.B.) were independently involved in this process. Any disagreement between the two authors was resolved through the involvement of a third author (D.M-G.), who acted as the final referee.

## Results

**Figure 2:**
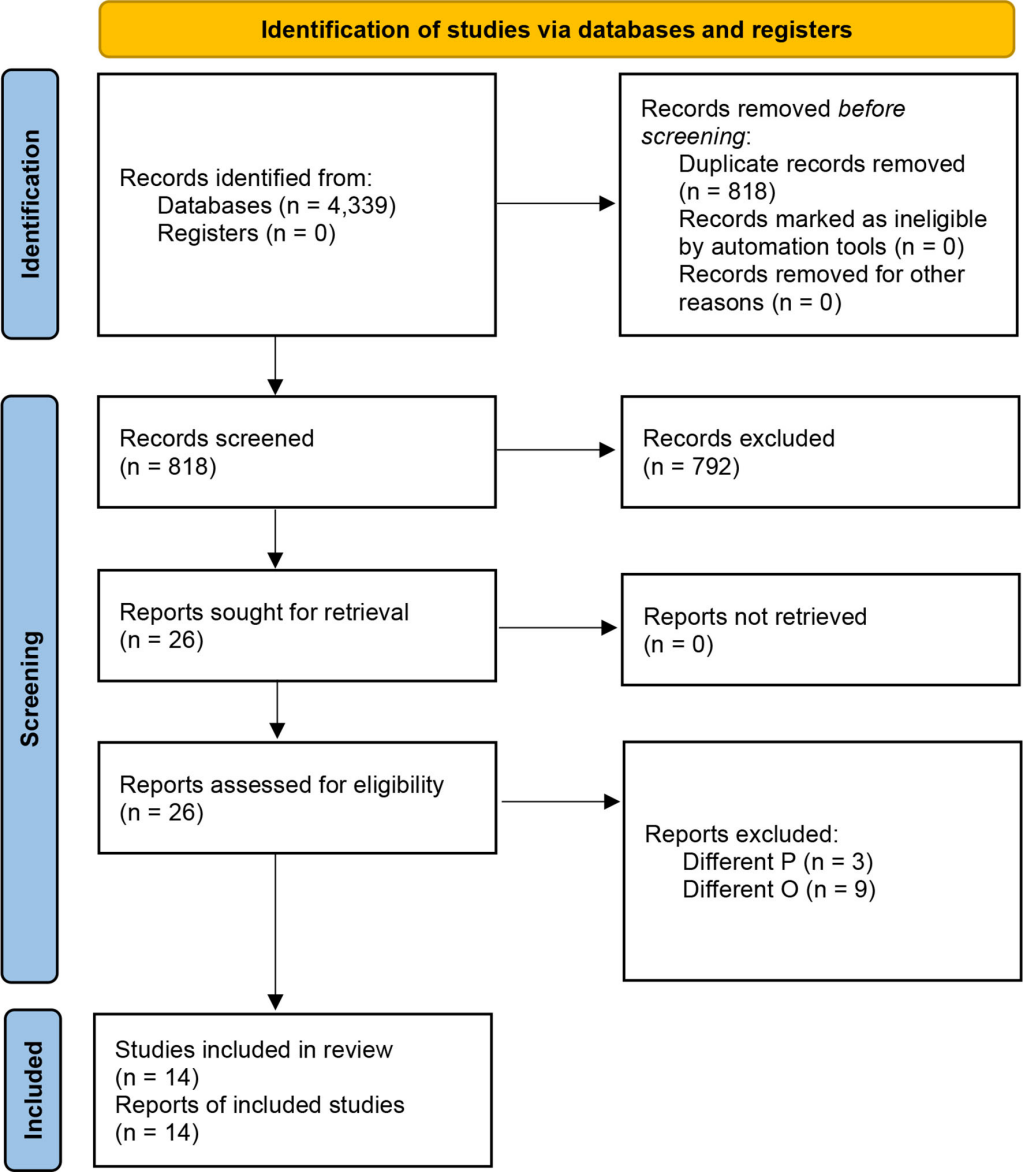
The PRISMA flowchart.

The initial list of retrieved items consisted of 4,339 studies (**Supplementary Table 1**). After the automated removal of 3,521 duplicates, 818 items were analyzed. Based on titles/abstracts, 792 studies were excluded and 26 full-texts were in-depth reviewed. Based on the above-mentioned inclusion/exclusion criteria, fourteen studies were included in the present systematic review (22–35) (**Figure 2**). Their major characteristics are reported in **Table 2**. The quality appraisal is shown in **Table 3** and **Supplementary Tables 3**-**5**.

**Table 2:** Major characteristics of studies included in the present systematic review.

Study	Study design	Country	Study period	Sample size	Main findings
Conic et al. (36)	Case-control, database-based study coupled with observational study	USA	From inception up to 2018	22,220 + 75 PsA patients	Red cell distribution width and mean platelet volume were predictors of major cardiovascular events in PsA patients
Costa et al. (37)	Observational study	Italy	During the COVID-19 pandemic (from 9 March 2020, for seven weeks)	105 PsA patients	Telemedicine services were well-accepted by PsA patients
Fagni et al. (38)	Review	NA	NA	NA	eHealth tools like JPAST can collect PROMs and combine them with biological (serological and genetic) data, potentially identifying early onset PsA
Gladman et al. (26)	Review/overview	Worldwide	NA	NA	A number of PsA-related registries were identified and an international model of dedicated registry was proposed
Gottlieb et al. (34)	ML and meta-analysis of 4 Phase 3 trials	NA	NA	2,148 PsA patients	ML identified predictors of response to secukinumab (“theratypes”)
Jalali−najafabadi et al. (39)	Observational study coupled with a database-based study	UK	NA	1,462 + 1,187 PsA patients	HLA_C_*06 and HLA_B_*27 were found to be the most important genetic features AUC ranged from 0.53 to 0.61
Love et al. (32)	Retrospective, database-based study	USA	1995-2007	2,318 PsA patients	31 PsA-related predictors could be identified by means of NLP and RF
Mc Ardle et al. (40)	Observational study based on serum proteomics coupled with multivariate machine learning analyses	Ireland	NA	32 + 95 PsA patients	AUC ranged from 0.69 to 0.94
Mulder et al. (41)	Observational study	The Netherlands	NA	41 PsA patients	ML can identify PsA inflatypes (with an AUC of 0.95)
Navarini et al. (33)	Observational study	Italy	NA	155 PsA patients	ML outperformed with respect to classical risk calculators in identifying cardiovascular endotypes
Ogdie et al. (27)	Retrospective cohort, claims database-based study	USA	2006-2019	13,661 patients	Higher percentage of complaints for arthritis and dermatological issues in PsA patients Arthritis, axial symptoms, and tendonitis/enthesitis generally preceded the diagnosis of PsA
Patrick et al. (42)	ML-based cohort study	NA	NA	Six cohorts with more than 7,000 genotyped PsA and psoriatic patients	Nine novel *loci* for psoriasis and its clinical subtypes were identified Biomarkers achieved an AUC of 0.82
Pournara et al. (43)	Observational study	USA	NA	1,894 PsA patients treated with secukinumab	Seven patient clusters (“endotypes” and “theratypes”) could be identified in terms of different articular, entheseal and cutaneous burden and therapeutic responses to secukinumab
Uhrenholt et al. (44)	Randomized, cross-over, observational study	Denmark	April 2019	20 PsA patients	Touchscreen devices and smartphone apps were well-accepted by PsA patients

AUC, area under the curve; ML, machine learning; NLP, natural language processing; PROMs, patient-reported outcome measures; PsA, psoriatic arthritis; RF, random forest.

NA, not applicable.

**Table 3:** Methodological quality assessment of the studies retained in the present systematic literature review.

Study	Quality assessment (number of yes)
Conic et al. (36)	8/9
Costa et al. (37)	3/8
Fagni et al. (38)	6/6
Gladman et al. (26)	5/9
Gottlieb et al. (34)	7/9
Jalali−najafabadi et al. (39)	9/9
Love et al. (32)	9/9
Mc Ardle et al. (40)	9/9
Mulder et al. (41)	6/9
Navarini et al. (33)	4/9
Ogdie et al. (27)	3/9
Patrick et al. (42)	9/9
Pournara et al. (43)	8/9
Uhrenholt et al. (44)	8/8

### Exploiting Large Epidemiological Databases and Registries for Early Intercepting Psoriatic Arthritis Patients

Gladman et al. (22) have reviewed existing registries specifically focused on psoriatic patients, including registries based at the University of Toronto, in Toronto, Ontario, and at the Memorial University, in St John’s, Newfoundland in Canada, as well as in Leeds, Bradford and Bath from the UK. Other psoriatic arthritis-related registries are based in Sweden, in Cagliari, Sardinia, in Italy, and in New York, USA. The authors have also proposed a general structure of an international registry specifically dedicated to the collection of psoriatic arthritis cases, in such a way that the different national/local registries can communicate with each other and be easily combined, integrated and mined by researchers. These registries can contain also biological information about patients and provide details about the “molecular epidemiology” of psoriatic arthritis.

Ogdie et al. (23) conducted a retrospective cohort study and mined MarketScan claims data from 2006 to 2019, totaling a sample of 13,661 patients. Subjects suffering from psoriatic arthritis reported a history of complaints for arthritis and dermatological issues in a higher percentage with respect to those without psoriatic arthritis. Arthritis, axial symptoms, and tendonitis/enthesitis generally preceded the diagnosis of psoriatic arthritis. Generally, psoriasis was diagnosed six months before psoriatic arthritis, which was intercepted by rheumatologists, followed by general practitioners and dermatologists.

### Deploying Artificial Intelligence Coupled With Epidemiological Big Data for Early Intercepting, Treating and Managing Psoriatic Arthritis Patients

Artificial intelligence can significantly complement and add to expert clinical knowledge. Love et al. (24) extracted a cohort of 2,318 psoriatic arthritis patients from a large academic electronic medical record database (comprising of more than 1,350,000 adult patients). Out of these 2,318 subjects with psoriatic arthritis, 550 were randomly selected for an in-depth chart review and for building, training and validating an artificial based-algorithm. The authors combined structured (i.e., codified) data with unstructured (i.e., textual/narrative, free-text) data and exploited natural language processing (NLP). By integrating these two forms of data, Love and colleagues were able to identify 31 psoriatic arthritis-related predictors. The positive predictive value (PPV) of a single psoriatic arthritis code was 57%, with a 95% confidence interval ranging from 55% to 58%. Integrating structured and unstructured data, by means of NLP, the predictive accuracy of the random forest algorithm significantly improved, reaching a PPV of 90-93% and a sensitivity of 87%, with a much increased area under the curve (AUC).

Navarini et al. (25) harnessed artificial intelligence for predicting cardiovascular risk in psoriatic arthritis patients. The use of already existing validated tools, such as the “Framingham Risk Score” calculator suffers from limitations. The authors explored the implementation of new *ad hoc* algorithms based on supervised machine learning techniques, such as support vector machine, random forest/feature analysis, and K-nearest neighbor. Machine learning approach proved to be feasible in psoriatic arthritis patients and outperformed with respect to conventional cardiovascular risk factor calculators. AUC ranged from 0.76 to 0.85.

Gottlieb et al. (26) coupled machine learning with evidence-based medicine and individual patient efficacy meta-analysis (IPEM). Authors deployed Bayesian elastic net to quantitatively evaluate baseline data from a cohort of 2,148 psoriatic arthritis patients, assessing a set of 275 predictors. Machine learning was able to identify predictors for an additional benefit of secukinumab.

### Deploying Artificial Intelligence Coupled With Molecular Big Data for Early Intercepting Psoriatic Arthritis Patients

Jalali−najafabadi et al. (27) exploited genetic/genomics data from 1,462 psoriatic arthritis and 1,132 psoriatic patients and applied a set of seven supervised artificial intelligence-based algorithms. Besides training and internally validating the risk prediction model, this was externally validated on a UK Biobank dataset consisting of 1,187 participants. HLA_C_*06 and HLA_B_*27 were found to be the most important genetic features. AUC was moderate, ranging from 0.53 to 0.55, which was slightly improved when adding further HLA features (AUC from 0.57 to 0.61).

Patrick et al. (28) devised an automated computational pipeline for predicting the insurgence of psoriatic arthritis among psoriasis patients utilizing data from six cohorts with more than 7,000 genotyped psoriatic arthritis and psoriatic patients. The authors were able to identify nine novel *loci* for psoriasis and its clinical subtypes, achieving a satisfactory AUC of 0.82 when combining a molecular signature consisting of 200 genetic markers. Precision and specificity were excellent (more than 90% and 100%, respectively), deploying conditional inference forest or shrinkage discriminant analysis.

Conic et al. (29) investigated the feasibility of exploiting the red cell distribution width and mean platelet volume as predictors of major cardiovascular events in psoriatic arthritis patients. The authors coupled a Big Data-based database (Explorys) with a smaller observational trial, to validate their findings. Higher values of the parameters under study predicted a higher cardiovascular risk as well as a poor therapeutic response.

Mulder et al. (30) aimed to detect disease-specific immune profiles from the phenotype of peripheral blood immune cells, which were effectively able to differentiate between psoriasis and psoriatic arthritis patients. Authors utilized a random forest-based algorithm coupled with in-depth flow cytometry and with an excellent AUC of 0.95 and found that psoriatic arthritis patients exhibited upregulated differentiated CD4+CD196+CD183-CD194+ and CD4+CD196-CD183-CD194+ T-cells, whereas CD196+ and CD197+ monocytes, memory CD4+ and CD8+ T-cell subsets and CD4+ regulatory T-cells were downregulated. Joint scores were found to correlate with memory CD8+CD45RA-CD197- effector T-cells and CD197+ monocytes.

Mc Ardle et al. (31) coupled cutting-edge serum proteomics with multivariate machine learning analyses to differentiate between patients suffering from psoriatic arthritis and those with rheumatoid arthritis. Different proteomics techniques were utilized: namely, nano-liquid chromatography mass spectrometry, SomaScan, an aptamer-based assays, and Luminex, a multiplexed antibody assay. AUC was ranging from 0.69 to 0.94, being the lowest for the bead-based immunoassay and the highest for the nano-liquid chromatography mass spectrometry. Machine learning applied on a subset of identified proteins could distinguish between psoriatic arthritis and rheumatoid arthritis patients with an AUC in the range of 0.79-0.85.

Finally, Pournara et al. (32) applied finite mixture models methodology on a cohort of 1,894 psoriatic arthritis patients treated with secukinumab to identify clinically meaningful clusters and phenotypes. The authors were able to identify seven patient clusters (“endotypes” and “theratypes”) in terms of different articular, entheseal and cutaneous burdens and therapeutic responses to secukinumab.

### Deploying Digital and Smart Technologies for Early Intercepting Psoriatic Arthritis Patients

In Denmark, Uhrenholt et al. (33) assessed the feasibility of collecting patient-reported outcome measures (PROMs) by means of smartphone apps. The authors find a high acceptability of touchscreen devices among rheumatological patients, including those suffering from psoriatic arthritis, with a high reliability of data collected and correlation with already validated measures of psoriatic arthritis.

Fagni et al. (34) reviewed the applications in the field of mHealth for early intercepting psoriatic arthritis patients. In particular, the authors identified the “Joint Pain Assessment Scoring Tool” (JPAST), a European Union-funded project on digital health, as a promising digital prognostic program that integrates PROMs (patient symptom checker inputs) with biological (serological and genetic) data.

Finally, Costa et al. (35) evaluated telemedicine services offered to a cohort of 105 psoriatic arthritis patients during the still ongoing “Coronavirus Disease 2019” (COVID-19) pandemic. Patients were willing to interact with their patients by means of live video-calls or telephone calls, could upload online photographs and pictures of their manifestations and could receive laboratory and/or instrumental exams and reports *via* email. During these services, pharmacological treatment could be added, switched or withdrawn/tapered.

## Discussion

To the best of our knowledge, this review represents the first systematic review addressing the current state of the use of Big Data, Artificial intelligence, digital and smart technologies for psoriatic arthritis. Most of the studies included concerned molecular applications of Big Data, especially in the fields of genomics and post-genomics. Other studies concerned epidemiological applications, with a practical dearth of studies assessing smart and digital applications for psoriatic arthritis patients.

Epidemiological/clinical Big Data can come from large-scale, often nationwide surveys. These data can guide public and global health policies as well as inform evidence-based medicine and, more specifically, dermatology and rheumatology on a variety of diseases, including psoriatic arthritis and collecting several related measures (39).

Whilst randomized controlled clinical trials represent the gold standard for building and appraising a body of rigorous and clinically relevant evidence, they are often time- and resource-consuming. may not always reflect real-life clinical practice and heterogeneous patient populations, as such limiting the generalizability and external validity of their findings. Real-life or real-world evidence, collected during daily clinical practice and by means of “pragmatic trials”, provides a complementary, “more relaxed” perspective to rigorously and tightly randomized controlled clinical trials (36, 42). While the latter are based on sometimes intrusive data collection methods, with regular study visits, the former exploit digital technologies (like smartphone applications, logs, and electronic health/medical records, or EHR/EMR). In this respect, Big Data-based studies can add to well-designed “small data”-based investigations and randomized controlled clinical trials (41).

To paraphrase what Doctor Lukas Kappenberger, pioneering father of the so-called “computational medicine”, has stated in 2005, the science (i.e., randomized controlled clinical trials) tells scholars and practitioners what they can do, the guidelines and checklists implement what they should do, and clinical registries/databases tell them what they are actually doing, and observing (40, 43).

Currently, there are lots of sources generating epidemiological Big Data, such as surveys, medical insurance data, vital registration data, cohort data, inpatient and outpatient data, among others (40).

These data can be retrospectively or prospectively collected: prospective clinical registries can be defined as large/very large datasets of observational data which have been collected prospectively and systematically and in a structured fashion, to reflect real-world clinical practices and outcomes of a given procedure (treatment, or surgical intervention) across large patient populations, including specific clinical/demographic (sub-)populations (40).

Furthermore, besides being complementary, randomized controlled clinical trials can be embedded within clinical registries (40): this enables to save time and resources and strengthens the generalizability of the findings (40).

Summarizing, Big Data repositories, registries, and databases are increasingly common in the field of rheumatological and dermatological practice and clinical research: there are, however, significant considerable variations in socio-demographic characteristics, co-morbidities, and major complication rates between individual (single- or multi-center) and database-based studies, and even among registry-studies themselves (for example, clinical versus administrative database). This should be accounted for when critically appraising rheumatological and dermatological research and in risk adjustment modeling (40).

In particular, administrative databases (40) can provide researchers and scholars, as well as practitioners and policy- and decision-makers with a lot of information concerning disease epidemiology, co-morbidities, disparities and inequalities in access to healthcare and clinical outcomes. Furthermore, they can inform in a data-driven fashion the decision-making processes underlying dermatological and rheumatological pharmacological treatments or surgical procedures, in terms of pre-operative risk stratification parameters in order to significantly curb/minimize peri-operative morbidity and mortality rates. On the other hand, administrative databases (40) may suffer from clerical inaccuracies, recording bias (due to the very nature of the database and secondary to economic-financial incentives underlying the collection, and maintenance of the dataset), temporal changes in nosology and nomenclature systems as well as in billing codes, and, finally, a dearth of several clinically relevant parameters, including rheumatological and dermatological specific variables and outcomes.

A major issue seriously limiting the deployment of databases and registries is related to their inter-operability and sometimes inconsistent use of definitions. Moreover, not all databases meet regulatory standards (40) and are enough curated/validated. As such, data standardization and meta-data are urgently warranted (40). In this sense, the proposal by Gladman et al. (22) is aimed at overcoming these issues.

Conversely, clinical studies, especially those relying on “Small Data”, even though well-designed and well-conducted, are generally statistically underpowered and are plagued by several biases, including participants sampling and selection bias, which hinders the generalizability of the findings, with samples being not representative of the entire population. It is also difficult to stratify according to a given dermatological or rheumatological pharmacological treatment or surgical procedure if the sample is particularly heterogeneous and the sample size does not allow to make sufficiently statistically robust and reliable calculations. Confidence and certainty can increase with “Big Data”, paralleling, however, the growth of complexity and associated computational costs (45, 46). Also, Big Data-based databases can be affected by biases, as previously mentioned, such as confounding, prevalent user bias (“the depletion of susceptibles”), immortal time bias, measurement bias, recording or association biases and other statistical artifacts, like “reverse epidemiology” or “reverse causality” (47). For instance, Escalante et al. (47), analysing a cohort of 779 patients with rheumatoid arthritis, found that body mass exerted a paradoxical effect on mortality, with patients with high body mass index reporting lower mortality. The authors mentioned that this paradoxical effect could be, at least partly, mediated by comorbidity and by the level of systemic inflammation. However, it is more plausible to deem this effect as a mere statistical artifact.

Furthermore, there are other issues plaguing Big Data-based databases, including selection bias (44). The so-called “index event bias”, which belongs to the family of selection biases, could explain what is known as the “risk factor paradox”, an unexpected phenomenon characterized by discrepant impacts of modifiable risk factors on the progression towards rheumatic diseases, such as osteoarthritis or rheumatoid arthritis.

Wet-lab, bioinformatics, and high-throughput technologies, including microarray chips, next-generation DNA and RNA sequencing and whole-exome sequencing, chromatin-immunoprecipitation-coupled sequencing, and mass-spectrometry-based proteomics analysis can generate a wealth of molecular big data, paving the way for a personalized/individualized rather than “one-size-fits-it-all” rheumatology and dermatology (38).

Molecular big data can elucidate the mechanisms underlying the etiopathogenesis of a given rheumatic/dermatological disease and identify new potential druggable targets for the development of *ad hoc* pharmacological therapies. Personalized rheumatology can benefit from genome-wide association and post-genomics studies (37), aimed at the identification of new transcription factors, genotypic and phenotypic validations of potential transcriptional regulators, and molecular/cellular mechanisms.

Summarizing, genomics and post-genomics (immunomics- and proteomics)-based assays coupled with machine learning could identify a set of biomarkers that could capture early psoriatic arthritis and differentiate it from other rheumatological conditions, providing individualized/personalized clinical subtype risk assessment. Once validated in larger cohorts, these panels could assist dermatologists and rheumatologists in their decision-making processes (37).

Latest technological achievements in the field of mobile health (mHealth) and ubiquitous health (uHealth), with smartphones, smart devices, smartwatches, and other wearable sensors (38) are revolutionizing the field of rheumatology and dermatology, directly involving, and engaging the patient, improving their therapeutical adherence and compliance, and also enabling remote patient monitoring. Wearable sensors of different types could enable the collection of different crucial parameters (37). Teledermatology and telerheumatology (48, 49) appear promising ways of early intercepting psoriatic arthritis patients.

To summarize, mHealth and digital health-based interventions, including telemonitoring or text messaging, can facilitate clinical data collection and can be customized, meeting the needs of “personalized rheumatology” and “personalized dermatology”.

## Conclusions and Future Prospects

Big data analytical techniques can be used to tackle dense, multidimensional datasets concerning clinically heterogeneous and complex diseases, like psoriatic arthritis. Contemporary dermatology and rheumatology can harness digital and smart technologies and artificial intelligence for early intercepting, treating and managing psoriatic arthritis patients. Big Data are increasingly having a more and more relevant role, being highly ubiquitous and pervasive in contemporary society, permeating it and paving the way for new, unprecedented perspectives in biomedicine, including rheumatology and dermatology. Big Data can be a real paradigm shift that revolutionizes dermatological and rheumatological practice and clinical research. However, there are some methodological issues that should be properly addressed and some ethical issues, including ensuring and preserving privacy, that should be considered. Therefore, further research in the field is urgently warranted.

## Data Availability Statement

The original contributions presented in the study are included in the article/**Supplementary Material**. Further inquiries can be directed to the corresponding author.

## Author Contributions

NLB conceived the paper. All other authors critically revised it. All authors contributed to the article and approved the submitted version.

## Funding

NLB was partially funded by the Celgene supported PARTNER fellowship programme.

## Conflict of Interest

The authors declare that the research was conducted in the absence of any commercial or financial relationships that could be construed as a potential conflict of interest.

## Publisher’s Note

All claims expressed in this article are solely those of the authors and do not necessarily represent those of their affiliated organizations, or those of the publisher, the editors and the reviewers. Any product that may be evaluated in this article, or claim that may be made by its manufacturer, is not guaranteed or endorsed by the publisher.

## Supplementary Material

Supplementary Table 1 Initial search literature results broken down according to each database mined.

Supplementary Table 2 Modified Qiao’s checklist for critically appraising Big Data/machine learning-based studies.

Supplementary Table 3 Outcomes of the assessment of the trustworthiness, relevance, and results of the studies retained in the present systematic literature review, based on the modified Qiao’s critical appraisal tool for critical methodological quality assessment of Big Data/machine learning-based studies.

Supplementary Table 4 Outcomes of the assessment of the trustworthiness, relevance, and results of the studies retained in the present systematic literature review, based on the Joanna Briggs Institute (JBI)’s critical appraisal tool for critical methodological quality assessment of cross-sectional investigations.

Supplementary Table 5 Outcomes of the assessment of the trustworthiness, relevance, and results of the studies retained in the present systematic literature review, based on the relevant Joanna Briggs Institute (JBI)’s critical appraisal tool for the critical methodological quality assessment (namely, the “Explanation of text and expert opinion critical appraisal tool”).
