# Supplementary material for: Harnessing Big Data, Smart and Digital Technologies and Artificial Intelligence for Preventing, Early Intercepting, Managing, and Treating Psoriatic Arthritis: Insights From a Systematic Review of the Literature
Source: Front Immunol. 2022 Mar 10;13:847312. doi: 10.3389/fimmu.2022.847312 (PMC8960164; doi:10.3389/fimmu.2022.847312)
Supplement: Supplementary Table 1 — Initial search literature results broken down according to each database mined. [file Table_1.docx]

**Supplementary Table 1. Initial search literature results broken down according to each database mined.**

| **Database** | **Number of items retrieved** |
| --- | --- |
| ProQuest Central | 584 |
| ProQuest Central UK/Ireland | 558 |
| Elektronische Zeitschriftenbibliothek - Frei zugängliche E-Journals | 450 |
| PubMed Central | 407 |
| IngentaConnect Journals | 368 |
| DOAJ Directory of Open Access Journals | 322 |
| MEDLINE (Ovid) | 295 |
| Journals@Ovid Ovid Autoload | 293 |
| ROAD: Directory of Open Access Scholarly Resources | 175 |
| Springer Online Journals Complete | 158 |
| Wiley Online Library Database Model 2020 | 120 |
| HighWire Press (Free Journals) | 104 |
| SpringerLINK- Lyrasis | 78 |
| Free Full-Text Journals in Chemistry | 77 |
| Springer Nature OA/Free Journals | 73 |
| Nature Journals Online | 69 |
| Wiley-Blackwell Open Access Titles | 63 |
| BioMedCentral Open Access | 60 |
| SpringerLINK Contemporary (FinELib) | 47 |
| Nature Open Access | 38 |

**Supplementary Table 2. Modified Qiao’s checklist for critically appraising Big Data/machine learning-based studies.**

| **Category/domain** | **Item** | **Explanations/details** | **Possible options** |
| --- | --- | --- | --- |
| Unmet need | Shortcomings, pitfalls and drawbacks affecting current non-Big Data/machine-learning approaches are properly discussed | These limits can be in terms of low diagnostic/prognostic accuracy, low predictive power, or due to particularly invasive, time- and resource-consuming procedures that may be difficult to implement in daily routine clinical practice | Yes/no |
| Reproducibility | Feature engineering methods/parameters choice | How features were generated before model training or how parameters and covariates were identified and selected | Yes/no |
|  | Platforms/packages | Both platforms/databases details and packages should be reported | Yes/no |
|  | Hyperparameters/meta-data | All hyperparameters/meta-data which are necessary for study replication by external researchers/research groups | Yes/no |
| Robustness | Valid methods to overcome over-fit | Use of reliable approaches and methods (such as leave-one-out or k-fold cross-validation or bootstrapping) to deal with potential over-fitting | Yes/no |
|  | The stability of the findings | Computed variation in the validation statistics | Yes/no |
| Generalizability | External data validation/replication | Validation/replication in settings/datasets/databases different from the research framework | Yes/no |
| Clinical significance | Predictors explanation | Explanation of the importance of each predictor/feature/covariate and its effect/impact on the outcome variable(s) | Yes/no |
|  | Suggested potential clinical uses and applications | Proposed potential applications to be implemented during daily routine clinical practice | Yes/no |

**Supplementary Table 3. Outcomes of the assessment of the trustworthiness, relevance, and results of the studies retained in the present systematic literature review, based on the modified Qiao’s critical appraisal tool for critical methodological quality assessment of Big Data/machine learning-based studies.**

| **Study** | **Unmet need** | **Reproducibility** | | | **Robustness** | | **Generalizability** | **Clinical significance** | |
| --- | --- | --- | --- | --- | --- | --- | --- | --- | --- |
| **Description** | **Limits in current approaches** | **Feature engineering/parameters choice** | **Platforms/ packages** | **Hyperparameter s/meta-data** | **Valid methods for over-fitting** | **Stability of results/sensitivity analysis** | **External data validation** | **Predictors explanation** | **Suggested clinical use** |
| Conic et al. [38] | Yes | Yes | Yes | Yes | No | Yes | Yes | Yes | Yes |
| Gladman et al. [26] | Yes | Yes | No | No | No | No | Yes | Yes | Yes |
| Gottlieb et al. [34] | Yes | Yes | No | No | Yes | Yes | Yes | Yes | Yes |
| Jalali‑najafabadi et al. [36] | Yes | Yes | Yes | Yes | Yes | Yes | Yes | Yes | Yes |
| Love et al. [32] | Yes | Yes | Yes | Yes | Yes | Yes | Yes | Yes | Yes |
| Mc Ardle et al. [40] | Yes | Yes | Yes | Yes | Yes | Yes | Yes | Yes | Yes |
| Mulder et al. [39] | Yes | Yes | No | No | Yes | No | Yes | Yes | Yes |
| Navarini et al. [33] | Yes | Yes | No | No | No | No | No | Yes | Yes |
| Ogdie et al. [27] | Yes | No | No | No | No | No | No | Yes | Yes |
| Patrick et al. [37] | Yes | Yes | Yes | Yes | Yes | Yes | Yes | Yes | Yes |
| Pournara et al. [41] | Yes | Yes | Yes | No | Yes | Yes | Yes | Yes | Yes |

**Supplementary Table 4. Outcomes of the assessment of the trustworthiness, relevance, and results of the studies retained in the present systematic literature review, based on the Joanna Briggs Institute (JBI)’s critical appraisal tool for critical methodological quality assessment of cross-sectional investigations.**

| **Study** | **Sample inclusion criteria** | **Study subject and setting** | **Exposure measurement** | **Objectivity of the exposure measurement** | **Identification of confounding factors** | **Confounding factors strategies** | **Objectivity of the outcome measurement** | **Appropriate use of statistical analysis** |
| --- | --- | --- | --- | --- | --- | --- | --- | --- |
| Costa et al. [47] | Yes | Yes | Yes | No | No | No | No | No |
| Uhrenholt et al. [45] | Yes | Yes | Yes | Yes | Yes | Yes | Yes | Yes |

**Supplementary Table 5. Outcomes of the assessment of the trustworthiness, relevance, and results of the studies retained in the present systematic literature review, based on the relevant Joanna Briggs Institute (JBI)’s critical appraisal tool for the critical methodological quality assessment (namely, the “Explanation of text and expert opinion critical appraisal tool”).**

| **Study** | **Opinion source** | **Standing in the field of expertise** | **Interests of the relevant population** | **Analytical process and logic** | **Reference to extant literature** | **Logically defended incongruence with the literature/source** |
| --- | --- | --- | --- | --- | --- | --- |
| Fagni et al. [46] | Yes | Yes | Yes | Yes | Yes | Yes |
